# Mechanical tuning of molecular machines for nucleotide recognition at the air-water interface

**DOI:** 10.1186/1556-276X-6-304

**Published:** 2011-04-07

**Authors:** Taizo Mori, Ken Okamoto, Hiroshi Endo, Keita Sakakibara, Jonathan P Hill, Satoshi Shinoda, Miki Matsukura, Hiroshi Tsukube, Yasumasa Suzuki, Yasumasa Kanekiyo, Katsuhiko Ariga

**Affiliations:** 1 World Premier International (WPI) Research Center for Materials Nanoarchitectonics (MANA), National Institute for Materials Science (NIMS), 1-1 Namiki, Tsukuba, Ibaraki 305-0044, Japan; 2 JST, CREST, Sanbancho, Chiyoda-ku, Tokyo, 1020075, Japan; 3 Osaka City University, 3-3-138 Sugimoto, Sumiyoshi-ku, Osaka, 558-8585, Japan; 4 Kitami Institute of Technology, 165 Koen-cho, Kitami, Hokkaido, 090-8507, Japan

## Abstract

Molecular machines embedded in a Langmuir monolayer at the air-water interface can be operated by application of lateral pressure. As part of the challenge associated with versatile sensing of biologically important substances, we here demonstrate discrimination of nucleotides by applying a cholesterol-armed-triazacyclononane host molecule. This molecular machine can discriminate ribonucleotides based on a twofold to tenfold difference in binding constants under optimized conditions including accompanying ions in the subphase and lateral surface pressures of its Langmuir monolayer. The concept of mechanical tuning of the host structure for optimization of molecular recognition should become a novel methodology in bio-related nanotechnology as an alternative to traditional strategies based on increasingly complex and inconvenient molecular design strategies.

## Introduction

**Figure 1:**
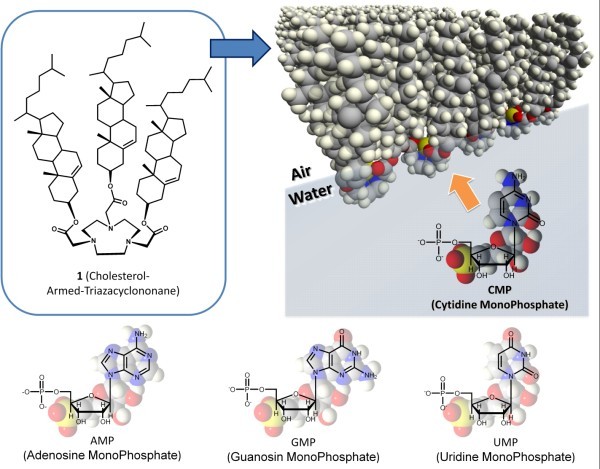
**Structures and schematic drawing of host 1 and guest nucleotides**.

Supramolecular structures constructed through bottom-up processes play crucial roles in nanoscience and nanotechnology [1,2]. In particular, those structures can be applied in bio-related nanotechnologies such as drug discrimination. Molecular assemblies immobilized at the air-water interface are appropriate media for incorporation of the sensing and diagnostic modules of aqueous biological molecules, since they provide great opportunities for molecular recognition of water-soluble guests by designer hosts in an insoluble floating monolayer [3]. Enhanced binding efficiencies of host-guest recognition at the air-water interface are in accord with theoretical simulations [4,5] and are supported experimentally as seen in selective sensing of aqueous peptides [6-8]. We have recently applied the concept of nanotechnology to these interfacial molecular recognition systems by embedding molecular machines in a Langmuir monolayer at the air-water interface where their mechanical operation can be operated by compressive surface pressure applied laterally [9]. The morphologies of the molecular machines can be controlled by macroscopic mechanical forces, resulting in optimization of structure for molecular sensing. We have previously demonstrated the (i) capture and release of fluorescent molecules upon cavity closure-opening motions of molecular machines [10-13], (ii) control of enantioselective binding of amino acids upon twisting motion of molecular machines [14,15], and (iii) discrimination of single-methyl-group difference between nucleobases (thymine and uracil) by control of macroscopic lateral pressures [16]. In our next demonstration of the utility of host molecules at the air-water interface, we show discrimination of some naturally occurring nucleotides, which are important in biological activities such as energy storage and signal transduction, using cholesterol-armed-triazacyclononane (**1**) as a molecular machine (see Figure 1 for recognition system). Using this strategy, we were able to discriminate between several ribonucleotides based on the twofold to tenfold difference in their binding constants under optimized conditions.

## Experimental

Water used for the subphase was distilled using an Autostill WG220 (Yamato) and deionized using a Milli-Q Lab (Millipore). Its specific resistance was greater than 18 MΩ · cm. Spectroscopic grade chloroform (Wako Pure Chemical Co., Osaka, Japan) was used as the spreading solvent. Ribonucleotides [adenosine 5'-monophosphate disodium salt (AMP), cytidine 5'-monophosphate disodium salt (CMP), guanosine 5'-monophosphate disodium salt (GMP), and uridine 5'-monophosphate disodium salt (UMP)] and lithium chloride were purchased from Wako Pure Chemical Co. (Osaka, Japan). The synthesis of the molecular machine, cholesterol-armed-triazacyclononane (**1**), was described previously [16]. Isotherms of surface pressure and molecular area (*π*-*A *isotherm) were measured at 20.0°C using an FSD-300 computer-controlled film balance (USI System, Fukuoka, Japan). A period of 15 min was allowed for spreading solvent evaporation, compression was commenced at a rate of 0.2 mm s^-1^. Fluctuation of the subphase temperature was within ± 0.2°C.

## Results and discussion

**Figure 2:**
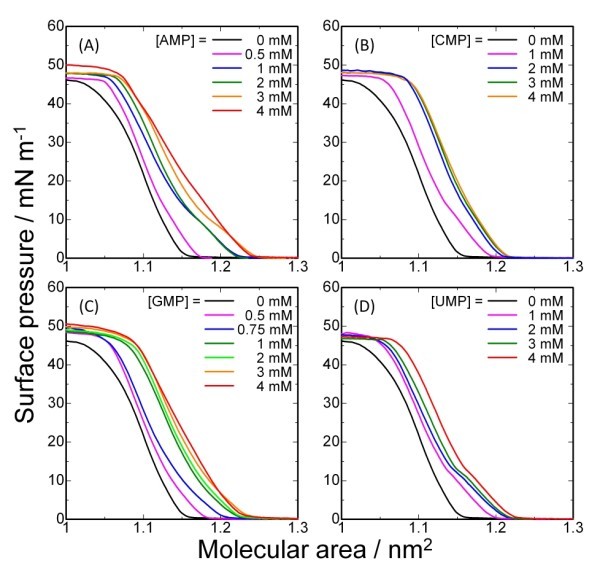
***π-A*****Isotherms of 1 with guests at 20°C without addition of LiCl into subphase:** (A) AMP; (B) CMP; (C) GMP; (D) UMP.

**Figure 3:**
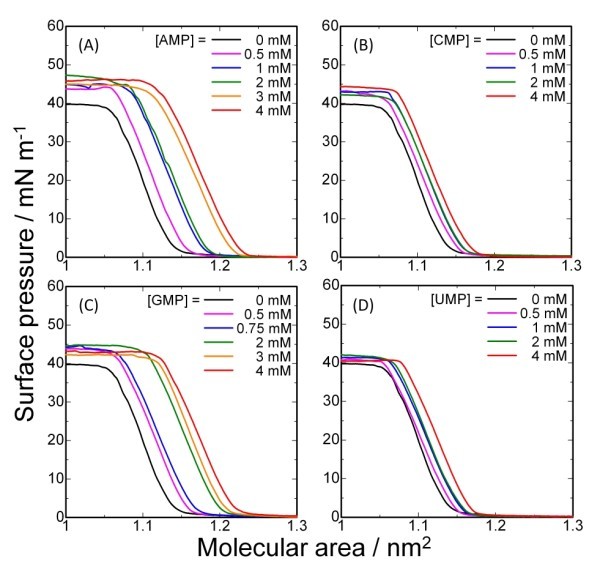
***π-A *Isotherms of 1 with guests at 20°C with 10 mM of LiCl**: (A) AMP; (B) CMP; (C) GMP; (D) UMP.

**Figure 4:**
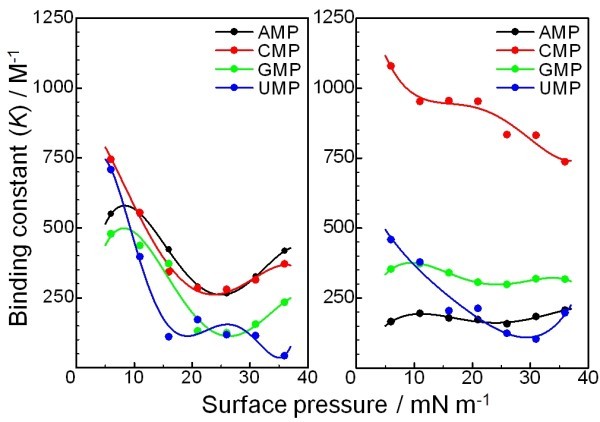
**Binding constant (*K*) of AMP, CMP, GMP, and UMP to the monolayer of 1 at various surface pressures at 20°C**: (A) without LiCl and (B) with 10 mM of LiCl.

*π-A *isotherms of the molecular machine **1 **with four different ribonucleotides (AMP, CMP, GMP, and UMP) in the subphase are shown in Figure 2 (on pure water) and Figure 3 (on aqueous solution of [LiCl] = 10 mM). In general, isotherms of **1 **under each condition exhibit monotonic increases without phase transitions. Increase in the nucleotide concentration in the subphase shifted the isotherms to larger molecular areas, suggesting that the molecular packing of **1 **was disturbed by interaction between the nucleotides and **1 **at the air-water interface. According to a reported method [14,16], the shifts in molecular areas at various guest concentrations can be converted into the binding constants (*K*) of nucleotides to the monolayer of **1 **at each surface pressure. The calculated values are summarized in Figure 4. In all the cases, assumption of an equimolecular binding gave the best fitting of the binding curves.

As shown in Figure 4A, the binding constants of the nucleotides to the monolayer of **1 **gradually decreased as the surface pressure increased. This is because expansion of the molecular area of **1 **by binding to the nucleotides is thermodynamically unfavourable at higher pressures. As will be described later, when the triazacyclononane moiety is not complexed with a central Li^+ ^ion electrostatic interaction between **1 **and the phosphate group within the nucleotide becomes less important. Hence, on the surface of pure water, there exists a rather ambiguous interaction between **1 **and the base portion of the nucleotides, and this interaction is quite sensitive to other factors. Although the absolute value of binding constants decreased drastically, differences in the binding efficiencies amongst the nucleotides became obvious at higher surface pressures. For example, ratios of binding constants, *K*(AMP/UMP), *K*(CMP/UMP), and *K*(GMP/UMP), are 0.78, 1.05, and 0.68, respectively, at a surface pressure of 5 mN m^-1^, whereas *K*(AMP/UMP), *K*(CMP/UMP), and *K*(GMP/UMP) values become 9.89, 8.77, and 5.52, respectively, when compressed to 35 mN m^-1^. Thus, discrimination of GMP and UMP from AMP and CMP is possible as well as between GMP and UMP, although differentiation between AMP and CMP is rather difficult even at greater surface pressures.

Complexation of Li^+ ^ion by the triazacyclononane ring causes two variations in the characteristics of the recognition system. The presence of Li^+ ^ion at the core of **1 **ensures strong electrostatic interaction between the monolayer and the nucleotides. In addition, the complexation of Li^+ ^ion stabilizes the conformation of the cyclononane ring of **1**, resulting in a rather simple situation of discrimination amongst the nucleotides (Figure 4B). Although the binding constants of UMP to the monolayer exhibit a distinct dependence on surface pressure, an order of binding constant (*K*(CMP) >*K*(GMP) >*K *(AMP)) is maintained over the entire pressure range. An apparent advantage in the Li^+^-containing system is due to a significant increase in the binding constant of CMP. As seen in Figure 3B, binding of CMP to the monolayer of **1 **does not require large expansion of the monolayer in contrast to AMP and GMP (Figure 3A, C). This binding mode should provide more favourable binding to the molecular assembly. On the other hand, the binding curve for UMP is unusual when compared with the other nucleotides. As has been suggested in previous research [16], the uridine moiety of UMP probably interacts with the cyclononane ring, thus competing with the major interactions between the phosphate and the Li^+ ^ion.

These results clearly indicate that the recognition of aqueous nucleotides can be tuned both by the surface pressure and the presence of Li^+ ^ion, although the same recognition element (**1**) was used throughout this investigation. The optimum discrimination between nucleotides can be obtained as follows, where the maximum ratio of binding constants and the conditions applied are summarized: *K*(CMP/AMP) = 6.5 ([Li^+^] = 10 mM and *π *= 5 mN · m^-1^); *K*(CMP/GMP) = 3.11 ([Li^+^] = 10 mM and *π *= 20 mN · m^-1^); *K*(CMP/UMP) = 8.77 ([Li^+^] = 0 mM and *π *= 35 mN · m^-1^); *K*(AMP/GMP) = 2.22 ([Li^+^] = 0 mM and *π *= 20 mN · m^-1^); *K*(AMP/UMP) = 9.89 ([Li^+^] = 0 mM and *π *= 35 mN · m^-1^); *K*(GMP/UMP) = 5.52 ([Li^+^] = 0 mM and *π *= 35 mN · m^-1^). On the other hand, the maximum binding constants for individual nucleotides are: *K*(CMP) = 1080 M^-1 ^([Li^+^] = 10 mM and *π *= 5 mN · m^-1^); *K*(AMP) = 550 M^-1 ^([Li^+^] = 0 mM and *π *= 5 mN · m^-1^); *K*(GMP) = 480 M^-1 ^([Li^+^] = 0 mM and *π *= 5 mN · m^-1^); *K*(UMP) = 710 M^-1 ^([Li^+^] = 0 mM and *π *= 5 mN · m^-1^). Therefore, conditions suitable for discrimination of the nucleotides and for most efficient binding of a single nucleotide component can be selected. The molecular recognition system presented here is therefore distinct different from more conventional ones where the structure of recognition components primarily defines binding efficiency of guest molecules.

## Conclusions

Prior to this and our other preliminary reports, discrimination of nucleotides has not been easy to achieve because of their structural similarity, and despite its importance in biological and pharmaceutical fields. This research strikingly demonstrates a method for molecular discrimination amongst structurally similar nucleotides by mechanical tuning of a simple host at a dynamic interfacial medium. Recognition and discrimination of ribonucleotides can also be optimized. The concept of mechanical tuning for optimization of molecular recognition should become a novel methodology in bio-related nanotechnology as an alternative to traditional strategies based on increasingly complex and inconvenient molecular design strategies.

## Abbreviations

AMP: adenosine 5'-monophosphate disodium salt; CMP: cytidine 5'-monophosphate disodium salt; GMP: guanosine 5'-monophosphate disodium salt; UMP: uridine 5'-monophosphate disodium salt.

## Competing interests

The authors declare that they have no competing interests.

## Authors' contributions

SS, MM, and HT carried out syntheses of molecular machines. TM, KO, HE, KS, JPH, YS, YK, and KA evaluate molecular recognition at the air-water interface. All authors read and approved the final manuscript.
